# (1*E*,4*E*)-1,5-Bis(2,6-difluoro­phen­yl)penta-1,4-dien-3-one

**DOI:** 10.1107/S1600536811007070

**Published:** 2011-03-02

**Authors:** Jun-Da Huang, Qin-Qin Tang, Xiao-Yan Chen, Yun Ye, Yi Wang

**Affiliations:** a School of Pharmacy, Wenzhou Medical College, Wenzhou, Zhejiang Province 325035, People’s Republic of China; b The First Affiliated Hospital, Wenzhou Medical College, Wenzhou, Zhejiang Province 325035, People’s Republic of China

## Abstract

The mol­ecule of the title compound, C_17_H_10_F_4_O, is roughly planar, with a dihedral angle of 5.59 (14)° between the two phenyl rings. The mol­ecule has an *E* conformation with respect to the olefinic bonds. In the crystal, mol­ecules are connected through C—H⋯O hydrogen bonds and there is slipped π–π stacking [centroid–centroid distance = 3.7983 (18), slippage =1.309 ;Å] between symmetry-related benzene rings.

## Related literature

The title compound is a derivative of the biologically active compound curcumin [systematic name (1*E*,6*E*)-1,7-bis­(4-hy­droxy-3-meth­oxy­phen­yl)-1,6-hepta­diene-3,5-dione]. For the biological activity and applications of curcumin, see: Aggarwal *et al.* (2007 ▶); Kamat *et al.* (2009 ▶); Liang *et al.* (2009 ▶); Pan *et al.* (1999 ▶); Sharma *et al.* (2007 ▶); Zhao *et al.* (2010*a* ▶,*b* ▶). For related structures, see: Zhao *et al.* (2009 ▶); Liang *et al.* (2007 ▶).
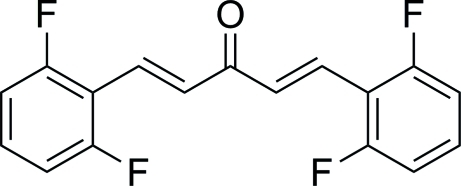


## Experimental

### 

#### Crystal data

C_17_H_10_F_4_O
*M* *_r_* = 306.25
Monoclinic, 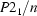

*a* = 7.7522 (11) Å
*b* = 15.413 (2) Å
*c* = 12.2848 (17) Å
β = 106.194 (2)°
*V* = 1409.6 (3) Å^3^
*Z* = 4
Mo *K*α radiation
μ = 0.13 mm^−1^
*T* = 293 K
0.40 × 0.37 × 0.23 mm

#### Data collection

Bruker SMART CCD area-detector diffractometer
Absorption correction: multi-scan (*SADABS*; Bruker, 2002 ▶) *T*_min_ = 0.640, *T*_max_ = 1.000
7288 measured reflections
2622 independent reflections
1612 reflections with *I* > 2σ(*I*)
*R*_int_ = 0.111

#### Refinement

*R*[*F* ^2^ > 2σ(*F* ^2^)] = 0.063
*wR*(*F* ^2^) = 0.174
*S* = 0.94
2622 reflections
199 parameters
H-atom parameters constrained
Δρ_max_ = 0.27 e Å^−3^
Δρ_min_ = −0.24 e Å^−3^

### 

Data collection: *SMART* (Bruker, 2002 ▶); cell refinement: *SAINT* (Bruker, 2002 ▶); data reduction: *SAINT*; program(s) used to solve structure: *SHELXS97* (Sheldrick, 2008 ▶); program(s) used to refine structure: *SHELXL97* (Sheldrick, 2008 ▶); molecular graphics: *SHELXTL* (Sheldrick, 2008 ▶) and *PLATON* (Spek, 2009 ▶); software used to prepare material for publication: *SHELXTL*.

## Supplementary Material

Crystal structure: contains datablocks I, global. DOI: 10.1107/S1600536811007070/dn2659sup1.cif

Structure factors: contains datablocks I. DOI: 10.1107/S1600536811007070/dn2659Isup2.hkl

Additional supplementary materials:  crystallographic information; 3D view; checkCIF report

## Figures and Tables

**Table 1:** Hydrogen-bond geometry (Å, °)

*D*—H⋯*A*	*D*—H	H⋯*A*	*D*⋯*A*	*D*—H⋯*A*
C16—H16⋯O1^i^	0.93	2.38	3.307 (4)	171
C8—H8⋯O1^ii^	0.93	2.39	3.308 (3)	170

Symmetry codes: (i) 
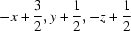
; (ii) 
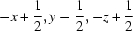
.

**Fig. 1.**
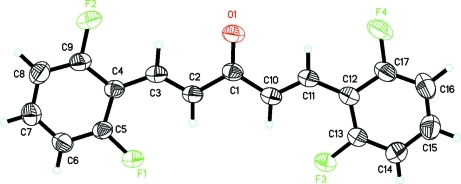
The molecular structure of the title compound with the atom labeling scheme. Displacement ellipsoids are drawn at the 30% probability level. Hydrogen atoms are represented as small spheres of arbitrary radii.

**Fig. 2.**
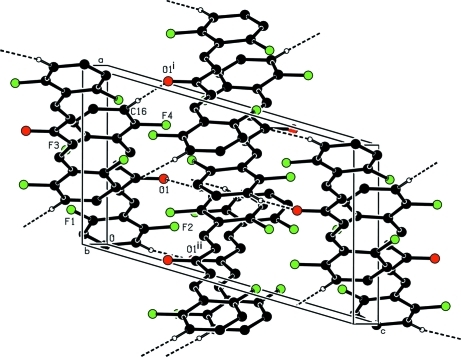
Partial packing view showing the C-H···O hydrogen bonds and the π-π stacking. H atoms not involved in hydrogen bondings have been omitted for clarity. Hydrogen bonds are shown as dashed lines. [Symmetry codes: (i) -x+3/2, y+1/2, -z+1/2; (ii) -x+1/2, y-1/2, -z+1/2]

## supplementary crystallographic information

### Comment

The title compound, (1E,4E)-1,5-bis (2,6-difluorophenyl) penta-1,4-dien-3-one (I), is one of mono-carbonyl analogues of curcumin designed and synthesized by our group.Curcumin (diferuloylmethane ) is the main component of turmeric, the powdered root of Curcuma longa *Linn*. Traditionally, curcumin has been used as a medicine for liver disease, indigestion, urinary tract diseases, rheumatoid arthritis, and insect bites (Aggarwal *et al.*, 2007; Kamat *et al.*, 2009). The pharmacological safety of curcumin has been demonstrated by its consumption for centuries at levels of up to 100 mg/day by people in certain countries (Pan *et al.*, 1999). One potential problem with the clinical use of curcumin is its low bioavailability and poor absorption characteristics ( Sharma *et al.*, 2007); however, curcumin remains an ideal leading compound for design of some effective analogues. In our previous study, a series of fluorine-containing, mono-carbonyl analogues of curcumin were designed and synthesized by the deletion of β-diketone moiety, and their bioactivities were evaluated (Liang *et al.*, 2009; Zhao *et al.*, 2010*a*,*b*). Among those compounds, some analogues exhibited better anti-tumor properties and a wider anti-tumor spectrum than curcumin. As a continuation of our broad program of work on the synthesis and structural study of curcumin analogues, the title curcumin derivative has been obtained and an X-ray diffraction study was carried out. Therefore, the structure of one of compounds (I), was further determined and analyzed using single-crystal X-ray diffraction. Accumulation of detailed structural and pharmacological data facilitated the explanation of the observed structure–activity relationships and modeling of new compounds with potential biological activity.

The molecule (I), consists of two 2,6-difluoophenyl rings linked through a penta-1,4-dien-3-one chain (Fig. 1). The molecule displays an *E* conformation with respect to the  olefinic bonds, exhibiting a butterfly-shaped geometry. The whole molecule is roughly planar with a dihedral angle between the two terminal phenyl rings of 5.59 (14)°. Among these derivatives, the structures of some of them were reported ( Liang *et al.*, 2007; Zhao *et al.*, 2009; Zhao *et al.*, 2010*a*,*b*).

In the crystal, the molecule are connected through C-H···O hydrogen bonds and slippest π-π stacking between symmetry related phenyl rings (Tables 1 and 2, Fig. 2).

### Experimental

Acetone (7.5 mmol) was dissolved in ethanol (5 ml) and crushed KOH (15 mmol) was added. The flask was immersed in a bath of crushed ice and a solution of 2,6-difluorobenzaldehyde (15 mmol) in ethanol (5 mmol) was added. The reaction mixture was stirred at 300 K and completion of the reaction was monitored by thin-layer chromatography. Ice-cold water was added to the reaction mixture after 48 h and the yellow solid that separated was filtered off, washed with water and cold ethanol, dried and purified by column chromatography on silica gel (yield: 49.3%). Single crystals of the title compound were grown in a CH_2_Cl_2_/CH_3_OH mixture (5:2 *v*/*v*) by slow evaporation .

Yellow powder, 49.3% yield, mp 135-138°C. ^1^H-NMR (CDCl_3_) δ: 6.95 (4H, t, Ar-H^3,5^×2), 7.29 (2H, d, *J*=18.0Hz, =CH-C=O×2), 7.31-7.36 (2H, m, Ar-H^4^×2), 7.81 (2H, d, *J*=18.0Hz, Ar-CH=C×2). ESI-MS *m/z*: 307.7 (M+1)^+^ 329.6 (M+Na) 635.3 (2M+Na), calcd for C_17_H_10_F_4_O: 306.25.

### Refinement

The H atoms were positioned geometrically (C—H = 0.93 and 0.96 Å) and refined as riding with *U*_iso_(H) = 1.2*U*_eq_(C) or 1.5*U*_eq_(methyl C).

### Crystal data

**Table:**

C_17_H_10_F_4_O	*F*(000) = 624
*M**_r_* = 306.25	*D*_x_ = 1.443 Mg m^−^^3^
Monoclinic, *P*2_1_/*n*	Mo *K*α radiation, λ = 0.71073 Å
Hall symbol: -P 2yn	Cell parameters from 1937 reflections
*a* = 7.7522 (11) Å	θ = 5.3–44.2°
*b* = 15.413 (2) Å	µ = 0.13 mm^−^^1^
*c* = 12.2848 (17) Å	*T* = 293 K
β = 106.194 (2)°	Prismatic, green
*V* = 1409.6 (3) Å^3^	0.40 × 0.37 × 0.23 mm
*Z* = 4	

### Data collection

**Table:**

Bruker SMART CCD area-detector diffractometer	2622 independent reflections
Radiation source: fine-focus sealed tube	1612 reflections with *I* > 2σ(*I*)
graphite	*R*_int_ = 0.111
φ and ω scans	θ_max_ = 25.5°, θ_min_ = 2.2°
Absorption correction: multi-scan (*SADABS*; Bruker, 2002)	*h* = −9→8
*T*_min_ = 0.640, *T*_max_ = 1.000	*k* = −10→18
7288 measured reflections	*l* = −13→14

### Refinement

**Table:**

Refinement on *F*^2^	Primary atom site location: structure-invariant direct methods
Least-squares matrix: full	Secondary atom site location: difference Fourier map
*R*[*F*^2^ > 2σ(*F*^2^)] = 0.063	Hydrogen site location: inferred from neighbouring sites
*wR*(*F*^2^) = 0.174	H-atom parameters constrained
*S* = 0.94	*w* = 1/[σ^2^(*F*_o_^2^) + (0.0971*P*)^2^] where *P* = (*F*_o_^2^ + 2*F*_c_^2^)/3
2622 reflections	(Δ/σ)_max_ < 0.001
199 parameters	Δρ_max_ = 0.27 e Å^−^^3^
0 restraints	Δρ_min_ = −0.24 e Å^−^^3^

### Special details

**Table:**

Geometry. All esds (except the esd in the dihedral angle between two l.s. planes) are estimated using the full covariance matrix. The cell esds are taken into account individually in the estimation of esds in distances, angles and torsion angles; correlations between esds in cell parameters are only used when they are defined by crystal symmetry. An approximate (isotropic) treatment of cell esds is used for estimating esds involving l.s. planes.
Refinement. Refinement of F^2^ against ALL reflections. The weighted R-factor wR and goodness of fit S are based on F^2^, conventional R-factors R are based on F, with F set to zero for negative F^2^. The threshold expression of F^2^ > 2sigma(F^2^) is used only for calculating R-factors(gt) etc. and is not relevant to the choice of reflections for refinement. R-factors based on F^2^ are statistically about twice as large as those based on F, and R- factors based on ALL data will be even larger.

### Fractional atomic coordinates and isotropic or equivalent isotropic displacement parameters (Å^2^)

**Table:**

	*x*	*y*	*z*	*U*_iso_*/*U*_eq_	
F1	0.1321 (2)	−0.05696 (10)	−0.13761 (11)	0.0953 (6)	
F2	0.2040 (3)	−0.14772 (13)	0.23505 (14)	0.1253 (7)	
F3	0.4725 (3)	0.31282 (12)	−0.12340 (13)	0.1193 (7)	
F4	0.7788 (3)	0.37861 (11)	0.25107 (15)	0.1277 (7)	
O1	0.4636 (3)	0.11929 (12)	0.21918 (15)	0.0902 (6)	
C1	0.4216 (3)	0.12103 (15)	0.1155 (2)	0.0650 (6)	
C2	0.3222 (3)	0.04845 (15)	0.0490 (2)	0.0651 (6)	
H2	0.2925	0.0506	−0.0297	0.078*	
C3	0.2739 (3)	−0.01982 (16)	0.09889 (19)	0.0665 (6)	
H3	0.3101	−0.0180	0.1777	0.080*	
C4	0.1752 (3)	−0.09625 (15)	0.05189 (18)	0.0625 (6)	
C5	0.1051 (3)	−0.11493 (15)	−0.06245 (19)	0.0654 (6)	
C6	0.0106 (3)	−0.18811 (17)	−0.1029 (2)	0.0761 (7)	
H6	−0.0336	−0.1970	−0.1806	0.091*	
C7	−0.0187 (4)	−0.24790 (18)	−0.0290 (3)	0.0816 (8)	
H7	−0.0837	−0.2979	−0.0561	0.098*	
C8	0.0473 (4)	−0.23481 (19)	0.0854 (3)	0.0908 (8)	
H8	0.0285	−0.2755	0.1368	0.109*	
C9	0.1404 (4)	−0.16122 (19)	0.1214 (2)	0.0793 (7)	
C10	0.4659 (3)	0.19589 (16)	0.0545 (2)	0.0672 (6)	
H10	0.4252	0.1973	−0.0242	0.081*	
C11	0.5625 (3)	0.26144 (16)	0.10904 (19)	0.0657 (6)	
H11	0.5979	0.2561	0.1876	0.079*	
C12	0.6217 (3)	0.33956 (16)	0.06650 (19)	0.0652 (6)	
C13	0.5821 (3)	0.36416 (17)	−0.0465 (2)	0.0776 (7)	
C14	0.6445 (4)	0.4382 (2)	−0.0837 (3)	0.0964 (9)	
H14	0.6124	0.4518	−0.1605	0.116*	
C15	0.7535 (4)	0.4915 (2)	−0.0071 (3)	0.0980 (9)	
H15	0.7977	0.5417	−0.0316	0.118*	
C16	0.7993 (4)	0.4721 (2)	0.1059 (3)	0.1009 (9)	
H16	0.8741	0.5086	0.1588	0.121*	
C17	0.7330 (3)	0.39840 (19)	0.1388 (2)	0.0832 (7)	

### Atomic displacement parameters (Å^2^)

**Table:**

	*U*^11^	*U*^22^	*U*^33^	*U*^12^	*U*^13^	*U*^23^
F1	0.1352 (13)	0.0939 (11)	0.0506 (8)	−0.0214 (9)	0.0155 (8)	0.0034 (7)
F2	0.1765 (17)	0.1274 (15)	0.0600 (10)	−0.0324 (12)	0.0129 (10)	0.0212 (9)
F3	0.1636 (15)	0.1215 (14)	0.0553 (9)	−0.0532 (12)	0.0018 (10)	0.0006 (9)
F4	0.1733 (17)	0.1124 (14)	0.0678 (11)	−0.0292 (12)	−0.0153 (10)	−0.0118 (10)
O1	0.1242 (15)	0.0820 (13)	0.0543 (11)	−0.0036 (10)	0.0084 (10)	−0.0012 (8)
C1	0.0676 (14)	0.0680 (15)	0.0553 (14)	0.0125 (11)	0.0102 (11)	−0.0008 (11)
C2	0.0719 (14)	0.0696 (15)	0.0512 (13)	0.0085 (11)	0.0131 (11)	−0.0007 (11)
C3	0.0693 (14)	0.0775 (17)	0.0506 (13)	0.0115 (12)	0.0134 (11)	0.0023 (12)
C4	0.0636 (13)	0.0656 (14)	0.0564 (14)	0.0099 (11)	0.0138 (10)	0.0024 (11)
C5	0.0739 (14)	0.0653 (15)	0.0564 (14)	0.0119 (12)	0.0175 (11)	0.0070 (11)
C6	0.0829 (16)	0.0755 (17)	0.0668 (16)	0.0058 (14)	0.0154 (13)	−0.0075 (13)
C7	0.0841 (17)	0.0689 (17)	0.093 (2)	0.0029 (13)	0.0272 (16)	−0.0040 (15)
C8	0.107 (2)	0.0768 (19)	0.093 (2)	0.0019 (16)	0.0336 (18)	0.0170 (16)
C9	0.0933 (18)	0.0828 (19)	0.0561 (15)	0.0050 (15)	0.0114 (13)	0.0086 (13)
C10	0.0671 (14)	0.0771 (17)	0.0525 (13)	0.0059 (12)	0.0088 (11)	−0.0050 (12)
C11	0.0675 (13)	0.0731 (16)	0.0518 (13)	0.0063 (12)	0.0087 (11)	−0.0052 (11)
C12	0.0629 (13)	0.0707 (16)	0.0588 (14)	0.0052 (11)	0.0118 (11)	−0.0061 (11)
C13	0.0811 (16)	0.0853 (18)	0.0611 (15)	−0.0131 (14)	0.0109 (12)	−0.0040 (13)
C14	0.111 (2)	0.096 (2)	0.0788 (19)	−0.0156 (18)	0.0214 (17)	0.0115 (16)
C15	0.099 (2)	0.082 (2)	0.112 (3)	−0.0120 (16)	0.0258 (19)	0.0076 (18)
C16	0.094 (2)	0.081 (2)	0.112 (3)	−0.0177 (17)	0.0032 (18)	−0.0135 (18)
C17	0.0870 (17)	0.0844 (19)	0.0655 (17)	−0.0005 (15)	0.0004 (13)	−0.0089 (14)

### Geometric parameters (Å, °)

**Table:**

F1—C5	1.342 (3)	C7—H7	0.9300
F2—C9	1.361 (3)	C8—C9	1.351 (4)
F3—C13	1.339 (3)	C8—H8	0.9300
F4—C17	1.360 (3)	C10—C11	1.323 (3)
O1—C1	1.224 (3)	C10—H10	0.9300
C1—C10	1.468 (3)	C11—C12	1.438 (3)
C1—C2	1.470 (3)	C11—H11	0.9300
C2—C3	1.323 (3)	C12—C13	1.388 (3)
C2—H2	0.9300	C12—C17	1.389 (3)
C3—C4	1.436 (3)	C13—C14	1.367 (4)
C3—H3	0.9300	C14—C15	1.353 (4)
C4—C5	1.388 (3)	C14—H14	0.9300
C4—C9	1.390 (4)	C15—C16	1.367 (4)
C5—C6	1.361 (3)	C15—H15	0.9300
C6—C7	1.357 (4)	C16—C17	1.354 (4)
C6—H6	0.9300	C16—H16	0.9300
C7—C8	1.370 (4)		
			
O1—C1—C10	121.1 (2)	F2—C9—C4	116.3 (2)
O1—C1—C2	120.5 (2)	C11—C10—C1	121.5 (2)
C10—C1—C2	118.4 (2)	C11—C10—H10	119.3
C3—C2—C1	121.3 (2)	C1—C10—H10	119.3
C3—C2—H2	119.3	C10—C11—C12	130.4 (2)
C1—C2—H2	119.3	C10—C11—H11	114.8
C2—C3—C4	130.9 (2)	C12—C11—H11	114.8
C2—C3—H3	114.6	C13—C12—C17	112.7 (2)
C4—C3—H3	114.6	C13—C12—C11	126.0 (2)
C5—C4—C9	112.6 (2)	C17—C12—C11	121.2 (2)
C5—C4—C3	126.3 (2)	F3—C13—C14	118.1 (2)
C9—C4—C3	121.1 (2)	F3—C13—C12	117.6 (2)
F1—C5—C6	118.1 (2)	C14—C13—C12	124.2 (3)
F1—C5—C4	117.8 (2)	C15—C14—C13	119.0 (3)
C6—C5—C4	124.1 (2)	C15—C14—H14	120.5
C7—C6—C5	119.4 (3)	C13—C14—H14	120.5
C7—C6—H6	120.3	C14—C15—C16	120.6 (3)
C5—C6—H6	120.3	C14—C15—H15	119.7
C6—C7—C8	120.2 (3)	C16—C15—H15	119.7
C6—C7—H7	119.9	C17—C16—C15	118.3 (3)
C8—C7—H7	119.9	C17—C16—H16	120.9
C9—C8—C7	118.2 (3)	C15—C16—H16	120.9
C9—C8—H8	120.9	C16—C17—F4	118.5 (3)
C7—C8—H8	120.9	C16—C17—C12	125.2 (3)
C8—C9—F2	118.1 (2)	F4—C17—C12	116.2 (3)
C8—C9—C4	125.5 (3)		
			
O1—C1—C2—C3	0.7 (3)	O1—C1—C10—C11	4.4 (3)
C10—C1—C2—C3	−178.2 (2)	C2—C1—C10—C11	−176.71 (19)
C1—C2—C3—C4	178.8 (2)	C1—C10—C11—C12	179.4 (2)
C2—C3—C4—C5	−0.4 (4)	C10—C11—C12—C13	2.2 (4)
C2—C3—C4—C9	179.5 (2)	C10—C11—C12—C17	−175.9 (2)
C9—C4—C5—F1	−179.4 (2)	C17—C12—C13—F3	−178.6 (2)
C3—C4—C5—F1	0.5 (3)	C11—C12—C13—F3	3.1 (4)
C9—C4—C5—C6	0.8 (3)	C17—C12—C13—C14	−0.2 (4)
C3—C4—C5—C6	−179.3 (2)	C11—C12—C13—C14	−178.5 (2)
F1—C5—C6—C7	179.9 (2)	F3—C13—C14—C15	179.2 (3)
C4—C5—C6—C7	−0.2 (4)	C12—C13—C14—C15	0.8 (5)
C5—C6—C7—C8	−0.3 (4)	C13—C14—C15—C16	−0.7 (5)
C6—C7—C8—C9	0.3 (4)	C14—C15—C16—C17	0.1 (5)
C7—C8—C9—F2	179.2 (2)	C15—C16—C17—F4	179.7 (3)
C7—C8—C9—C4	0.4 (4)	C15—C16—C17—C12	0.5 (5)
C5—C4—C9—C8	−0.9 (4)	C13—C12—C17—C16	−0.4 (4)
C3—C4—C9—C8	179.2 (2)	C11—C12—C17—C16	177.9 (3)
C5—C4—C9—F2	−179.7 (2)	C13—C12—C17—F4	−179.6 (2)
C3—C4—C9—F2	0.4 (3)	C11—C12—C17—F4	−1.3 (4)

### Hydrogen-bond geometry (Å, °)

**Table:**

*D*—H···*A*	*D*—H	H···*A*	*D*···*A*	*D*—H···*A*
C16—H16···O1^i^	0.93	2.38	3.307 (4)	171
C8—H8···O1^ii^	0.93	2.39	3.308 (3)	170

Symmetry codes: (i) −*x*+3/2, *y*+1/2, −*z*+1/2; (ii) −*x*+1/2, *y*−1/2, −*z*+1/2.

### Table 2 Table 2 π-π stacking interactions (Å)

Cg1 is the centroid of the C12-C17 ring.

**Table:**

CgI	CgJ	CgI···CgJ^a^	CgI···P(J)^b^	CgJ···P(I)^c^	Slippage
Cg1	Cg1^iii^	3.7983 (18)	3.5656 (12)	3.5656 (12)	1.309

Symmetry codes: (iii)1-x,1-y,-z Notes: a : Distance between centroids b : Perpendicular distance of CgI on ring plan J. c : Perpendicular distance of CgJ on ring plan I. Slippage = vertical displacement between ring centroids.
